# The art of retirement for orthopedic surgeons

**DOI:** 10.4103/0019-5413.53451

**Published:** 2009

**Authors:** Surendra Mohan Tuli

**Affiliations:** Vidhyasagar Institue of Mental Health and Allied Sciences, Lajapt Nagar, New Delhi – 110065

“But there's life in these old boots still, I tell you A flickering flame where once there was fire
But a flame, nonetheless
To more, I can no longer aspire”.

Albert Einstein: Retirement has a definitive but variable impact on an individual's life. The outcome of research, on impact of retirement on general population, has been inconsistent. While some studies show retirement accompanied by poorer health, more depression and loneliness, less life satisfaction and happiness, others documented a positive impact.^1^ There are, however, scanty reports on retirement of physicians in general and orthopaedic surgeons in particular. In general orthopaedics surgeons have a very hectic life schedule, with lots of patient care activities, and have plenty of opportunities to embrace happiness and satisfaction. They are in demand, as well satisfied, because of the gratitude they receive from patients, with and/or pain. The transition from a very active to not so active life may be sudden as, on a particular date, people working on any chair may retire or may be well organised and thoughtful and have a planned transition. The predictors of life satisfaction include good health, satisfactory income, good relationship with spouse and children and an increase in travel or vacation activity. It is important to plan retirement well so that satisfaction remains high making post retirement life enjoyable.

This write-up is based on a discussion which evolved in a session organized during the annual conference of the Indian Orthopaedic Association between December 2-7 2008, at Banglore. Dr. R.L. Mittal (Patiala) and Dr.Thomas Chandy (Bangalore) were panelists. The theme of discussion was “life after retirement”. The room,was filled with over 150 orthopaedic surgeons between the age group of of 40-70. Most of the discussion was initiated by younger participants. There was concern about the life after retirement, discussions on active surgical practice to the last days of existence. A few important practical suggestions that developed are summarized below.

Broadly speaking, the preparation for that phase of life must start (like pre-operative planning) at around 40 years. Whatever we sow at that age could be reaped at an older age.

## HEALTH AND LIFE STYLE

Good health is a necessity. Though most of us in the Indian subcontinent have inherited strong genes, our ancestors belong to the generation of the fittest survivors -, having survived cholera, plague, malaria, other infections, famine and deprivation. However, even the best of DNAs require healthy environments and a healthy life style. Early diagnosis and timely treatment can control most ailments except probably malignancies. Rapidly increasing life span would permit most of the clinicians to reach an average of 70 years or more. A study conducted on 708 retired orthopaedic surgeons of a mean age of 72.9 yrs, found that 85% had excellent or good health and 22% reported worse health than before retirement.^1^

The suggestions for healthy life include maintenance of your body mass (weight and waste line) with balanced food habits, abstinence from tobacco and no more than moderate use of alcohol and caffeine. About 35 minutes of whole body exercises would go a long way to strengthen our muscles, minimize osteoporosis, and maintain reflexes and falls. The habit of exercise should start from younger days and not around retirement. If done during active life it increases endurance while starting at 65 years, after retirement, will help in just maintaining tissue mass.

An average orthopaedic specialist is well aware of the importance of diet, exercise, controllable healthy life style factors, and enlightened societies. About 64 per cent of medical doctors can be expected to live up to at least 75 years and 35 per cent to at last 85 years. More recently, the latter half of 20^th^ century provided us effective treatment for cardiovascular diseases, diabetes, renal disease, arthritis and debilitating disorders like dementias and Alzheimer's.

What is important after 65 is to convert most of the “remaining years” to active life. It is particularly important to note that above 80 per cent of the elderly have at least one chronic health problem and 50 per cent have at last two. One should get attuned to cope with the limitations imposed.^2^

## FINANCIAL SECURITY

We get used to a particular life style during our active professional life. We would feel comfortable if one can maintain nearly the same life style after the active professional life. Medical insurance, long- term fixed deposits in bank, reasonable living accommodation with suitable ambience are minimum essential that should be provided. As we cross 60s, we may not be able to keep a track of our investments in stocks, banks and other locations. It is wise around 60s to consolidate investment in two or three locations to minimize the “forgotten assets”. Presently, available facilities of computer and keeping your spouse fully informed would safeguard your assets. We must be more organized to secure a regular “monthly pension” scheme, say through your job, at the age of 40 years. Adequate and sustained income is a pre-requisite for enjoyable and comfortable post-retirement life^3^. The financial planning for retirement must begin early. For retirement at 65 years, if a person starts planning around 35 years, he will have to save two and a half times less than if he starts at the age of 50 years. However, if retirement is delayed by another five years, it can come down to 50% more than what he saves with planning at 35 years.

The meeting of your retirement goal is easier when time is on your side. The power of compounding greatly reduces the amount of money that you actually have to save to meet your retirement goals if you begin early. Hence, financial planning for retirement must begin at the age of 40 years so that one saves enough money for a decent post-retirement life without curbing on financial expenditure during young age.^4^

## EMOTIONAL FAMILY STABILITY

One of my teachers Prof. Duraiswami advised me, “Tuli when you are going up the ladder, do not look down upon the person whom you cross. You will meet them again when you start coming down”. We should remember our heydays and maintain a good relationship with peers, students, colleagues, family members and spouse. We should be cognizant of the support we receive from spouse, children and other members of the family.

Fortunately, in most of the homes in the Indian subcontinent the “school of marriage and family” is quite strong and adaptive. A conscious effort must, however, be made to nurture this relationship for your own self as well as for the next generation. It is the spouse and your family who would give you company, share your thoughts and activities, and care for you willingly (of due to social obligation/ pressure) during the years beyond sixties. These relationships need to be cultured and nurtured again since 40 years of age, these are the most important and valuable assets you have.^5^ In a study, 25% orthopaedic surgeons (n is equal to 708) felt that they had more time to spend with their families. While 10% found this relationship more challenging. The surgeons who had less satisfactory relationship with their spouse before retirement, found it more challenging, post-retirement.

## LEISURE AND SOCIAL ACTIVITIES

You can become a member of social clubs, like rotary club, loins club etc. or other country clubs in your city and participate in regular meetings and get–togethers. As a clinician you can prepare and deliver talks on subjects of broader interested like, knee-pain, neck pain, low-back-pain, osteoporosis, bone health depending on your health, time and inclination and families, organized by these clubs or can participate in teaching sessions for post-graduates in the nearby medical school or some time in post-graduate training courses.

## MODIFIED CONTINUATION OF PROFESSIONAL AND CLINICAL ACTIVITIES

Being a life member of the Indian Orthopaedic Association and a member of one or two of super specialty societies (there are nearly a dozen of these) offers you an opportunity to attend their academic conferences. Delegation fees are thankfully exempted for senior citizen (above 65 years) at most of such meetings. Such meetings offer us a peek or glimpse of the excitement and activities of younger generation, a platform for intellectual interaction, meeting with long-time friends and contemporaries, social gathering of families, and visit to new places. As long you are ambulatory, these meetings are rewarding in many ways.

Mahatma Gandhi said, “Men and women approaching retirement age should be recycled for public service work, and their companies should foot the bill. We can no longer afford to scrap- pile people”. We can be involved in the activities of the resident Welfare Association we settle, share your thoughts with the people for preventive medicine, pollution and environment protection. Such pursuits give you sense of creativity. With the universal availability of internet facilities one can use this technology to create your own blog for self expression and interaction with other concerned people, join an internet medical consultation and internet teaching programs.

## TRANSCENDENTATION AND KARMA

We must strive (since the age of 40 years) to have an active post-retirement life, with least duration of time in bed prior to the final event of demise. We, however, do need prayers for such a blessing. Destiny or kismet may play a role for the comfort or otherwise around the last universal event. We as doctors pass through gruelling years of training, observation, success and failures. Some of us get influenced by vanity and false values. At every operative procedure we pray for good luck, even if we have not seen “god” we do perceive the role of cosmic power. Messengers of all faiths and religions have one common constant – the theory of karma, therefore let us strive throughout our active years to build up an assemblage of KARMAS to have peace and comfort in the culminating years on terra firma. Just like we build up our pension funds we also need to build up the store of spiritual and intellectual capital.

There are enjoyable aspects of retirement - one is able to spend more time for family, ability to travel and freedom from schedule and time constraint. The most challenging and difficult aspect of retirement is the loss of role as orthopaedic surgeon while the freedom from their schedule is more rewarding. In general, surgeons and academicians feel like strangers in the hospital. The lack of satisfaction of professional accomplishment or loss of prestige and loss of medical fraternity is the most difficult.^1^ Counselling is needed to face the loss of medical practice. There is a need to explore new professional interest before retirement and need to be sensitive to the interest and concerns for one's spouse and family both before and after retirement. One retired surgeon has said, one needs three things for successful retirement:

1. Enough money.
2. Outside interest (other than medicine).
3. Knowing in one's heart that one's self worth is not dependent on being a doctor or orthopedic surgeon.

If one has these three, one can retire any time at any age, happily.
